# Pure Air on the Sea

**Published:** 1878-02

**Authors:** 


PURE AIR OF THE SEA, but as a matter of fact very little of it is obtained by passengers as a general rule, because a bilgy odor pervades the cleanest ship's cabin, and when it is taken into account that in these cabins passengers confine themselves from sun down to a late breakfast next day, and that soon after breakfast the decks, having been washed, are still wet, making it near noon before it is safe for ladies, with their thin shoes, to promenade; it is evident that a very few hours of the most pleasant days are devoted to the breathing of the pure salt sea air, and when it is remembered too, how few days at sea have an entire exemption from rain and raw winds, it is evident the much lauded good effects of sea voyages, especially to invalids, is more a myth than a r[illegible]. The truth is, to obtain the very highest healthful advantages of pure air, nothing approaches moderate, leisure working in

THE GARDEN OR THE ORCHARD.

Next to that, especially for the ailing, is a continuous

